# Reparameterizable Multibranch Bottleneck Network for Lightweight Image Super-Resolution

**DOI:** 10.3390/s23083963

**Published:** 2023-04-13

**Authors:** Ying Shen, Weihuang Zheng, Feng Huang, Jing Wu, Liqiong Chen

**Affiliations:** College of Mechanical Engineering and Automation, Fuzhou University, Fuzhou 350108, China

**Keywords:** lightweight image super-resolution, reparameterizable multibranch bottleneck module, PSE loss, edge computing device

## Abstract

Deployment of deep convolutional neural networks (CNNs) in single image super-resolution (SISR) for edge computing devices is mainly hampered by the huge computational cost. In this work, we propose a lightweight image super-resolution (SR) network based on a reparameterizable multibranch bottleneck module (RMBM). In the training phase, RMBM efficiently extracts high-frequency information by utilizing multibranch structures, including bottleneck residual block (BRB), inverted bottleneck residual block (IBRB), and expand–squeeze convolution block (ESB). In the inference phase, the multibranch structures can be combined into a single 3 × 3 convolution to reduce the number of parameters without incurring any additional computational cost. Furthermore, a novel peak-structure-edge (PSE) loss is proposed to resolve the problem of oversmoothed reconstructed images while significantly improving image structure similarity. Finally, we optimize and deploy the algorithm on the edge devices equipped with the rockchip neural processor unit (RKNPU) to achieve real-time SR reconstruction. Extensive experiments on natural image datasets and remote sensing image datasets show that our network outperforms advanced lightweight SR networks regarding objective evaluation metrics and subjective vision quality. The reconstruction results demonstrate that the proposed network can achieve higher SR performance with a 98.1 K model size, which can be effectively deployed to edge computing devices.

## 1. Introduction

With the development of deep learning [1], single image super-resolution (SISR) models based on the convolutional neural network (CNN) [2] have achieved excellent performance compared to traditional interpolation methods. However, increasing network complexity is frequently used to improve reconstruction performance, making it challenging to deploy the latest super-resolution (SR) algorithms on resource-limited edge computing devices. On the one hand, although neural processor units (NPUs) optimized for neural network are common on edge computing devices, most SR networks do not consider compatibility with NPUs and hence cannot fully utilize NPUs. On the other hand, the requirement for high-resolution (HR) image processing (720 p/1080 p or higher) significantly increases memory and computational requirements. The existing SISR algorithms have very limited optimization for NPUs. Operations contained in the networks that are not supported by the NPUs will be partially processed by the CPUs or GPUs. This introduces additional data transfer costs between processors, resulting in a significant computational overhead. Therefore, there is growing interest in how to deploy SR models on resource-limited devices while improving computational efficiency.

Recently, many lightweight models have been proposed to facilitate the deployment [3,4]. Ahn et al. [5] proposed the cascading residual network (CARN), which reused information from different levels by cascading residuals, but the performance of this method significantly drops. Li et al. [6] designed a feedback network (SRFBN), which adopted a recurrent neural network structure to share the parameters of the hidden layers, but this method is not sufficiently lightweight and its performance needs further improvement. Hui et al. [7] proposed the information multidistillation network (IMDN), which employed a multiple information distillation block to distill and selectively fuse some features to compress parameters while enhancing the performance. Liu et al. [8] proposed the residual feature distillation network (RFDN), which built on IMDN by replacing all channel separation operations with 1 × 1 convolution and adding feature distillation connections to further improve the performance. These lightweight models can effectively reduce the number of parameters and floating-point operations (FLOPs). However, recent studies have shown that the number of parameters and FLOPs does not necessarily correlate positively with the performance on edge computing devices [9]. For example, residual connections and multibranch structures are commonly used in lightweight SISR tasks [10]. These operations may lead to high memory access costs, which hinder fast operation on edge computing devices. In addition, most of the existing efficient SISR networks have been evaluated for performance only on GPUs, which does not reflect their running speed on edge devices.

To minimize computational costs even more, reparameterization [11] is implemented into the SISR tasks. Zhang et al. [12] proposed an edge-oriented convolution block for real-time super-resolution (ECBSR), which provided high reconstruction quality while preserving fast inference speed by collapsing training multibranch modules into normal 3 × 3 convolutions in the inference phase. Bhardwaj et al. [13] proposed collapsible linear blocks for super-efficient super-resolution (SESR), which achieved 60 FPS reconstruction for 4K images on mobile devices by a folding network structure. However, both ECB and CLB fail to take full advantage of reparameterization and have some problems such as model overfitting, long training time, and slow convergence, which may limit the SR performance. To address these problems, we design a reparameterizable multibranch bottleneck module (RMBM) that consists of a bottleneck residual block (BRB), an inverted bottleneck residual block (IBRB), and an expand–squeeze convolution block (ESB). Based on these important components, RMBM can increase the field of perception and effectively improve model expression. RMBM accelerates the convergence of the model and solves the overfitting problem by using residual connection and normalization. Furthermore, we design a lightweight SISR model, termed the reparameterizable multibranch bottleneck network (RMBN), based on RMBM, which can reduce performance degradation caused by model quantization during deployment.

To stabilize the training and refine the parameters, SISR tasks often utilize L1 loss and L2 loss to determine the pixel disparities between the reconstructed images and ground truth. However, it has been proved that using a single loss is insufficient for accurately restoring locally varying diverse shapes in images, often generating undesirable artifacts or unnatural details [14]. To enhance the visual effect, Ledig et al. [15] proposed the super-resolution generative adversarial network (SRGAN), which used a succession of complicated losses. Nevertheless, the generative adversarial network (GAN) [16] training procedure is challenging and prone to gradient disappearance, collapse, and training instability. To address the issue of oversmoothed SR images, we introduce a simple but efficient peak-structure-edge (PSE) loss in this work. This new loss allows the network to concentrate more on the recovery of high-frequency texture details.

The main contributions of this work can be summarized as follows:

(1) We propose a lightweight image SR network, named RMBN, which uses the residual learning of image dimension and feature dimension to make the network focus on recovery of high-frequency information. Additionally, by adding constrained activation, the performance degradation of the uint8 model is decreased. The deployment SR network can run efficiently and stably on the edge device equipped with the rockchip neural processor unit (RKNPU).
(2) We propose RMBM to improve the expressiveness of the model by increasing the width and depth of the network during the training phase. In the deployment phase, RMBM is equivalently transformed into a simple convolutional layer using reparameterization to reduce the number of parameters.
(3) We propose a novel PSE loss that takes into account the recovery of both global and edge information and achieves better balance between perception quality and objective evaluation metrics.

The rest of this work is organized as follows. In Section 2, related works concerning CNN-based SISR methods and structure reparameterization are summarized. We also introduce model optimization in the same section. In Section 3, the network structure is proposed and the process of reparameterization is mentioned. Section 4 details the experimental results of the proposed method and compares it with state-of-the-art methods in natural image datasets and remote sensing image datasets. At last, the conclusions are drawn in Section 5.

## 2. Related Work

### 2.1. Single Image Super-Resolution

In a pioneer work, Dong et al. [17] proposed a shallow super-resolution CNN (SRCNN), which consisted of three layers of convolutional neural network and was used to perform end-to-end learning of image super-resolution. This approach showed outstanding performance compared to conventional solutions. Kim et al. [18] proposed very deep super-resolution (VDSR), which applied a residual learning strategy to SISR by increasing the network depth. Shi et al. [19] proposed an efficient subpixel convolutional neural network (ESPCN), which produced the subpixel operation, which is a learnable upsampling layer. Inspired by ESPCN, increasingly excellent SISR networks were proposed. Lim et al. [20] proposed an enhanced deep super-resolution network (EDSR), which integrated the modified residual blocks, considerably improving SISR performance. Other works, such as enhanced super-resolution generative adversarial network (ESRGAN) [21], persistent memory network (MemNet) [4], and residual dense network (RDN) [22], explored dense connectivity by using all features of the convolutional layers. Although these methods achieved significant performance, they were costly in memory consumption and computational complexity, limiting their applications on edge computing devices. Some recent SISR networks focus on the tradeoff between performance and complexity. CARN [5] used group convolution to make image SISR networks lightweight and efficient. Hui et al. [23] designed an information distillation network (IDN), which proposed a residual feature distillation structure for better exploiting hierarchical features. IMDN [7] improved IDN by using a channel-splitting strategy in an information multidistillation block. RFDN [8] rethought the channel splitting operation and introduced the progressive refinement module as an equivalent architecture. Different from other models, the linearly assembled pixel-adaptive regression network (LAPAR) [24] transformed SISR tasks to linear regression tasks for multiple base filters. Luo et al. [25] designed the lattice block network (LatticeNet), which used a lattice filter based on the butterfly structure and applied reverse fusion strategy to extract hierarchical context information. These works maintained good tradeoff between performance and model complexity on GPUs, but their performance on edge computing devices has to be studied further. Herein, we propose a single-branch deployment network consisting of simple operators that are suitable for most edge computing devices and can be run efficiently on NPUs.

### 2.2. Structure Reparameterization

The asymmetric convolution block network (ACNet) [11] was the first to apply the concept of structure reparameterization. It refers to the methodology that parameterizes a structure with the parameters transformed from another structure. Ding et al. [26] introduced a convolutional neural network architecture termed RepVGG, which was similar to the very deep convolutional network (VGG) [27]. It employed reparameterization to decouple the multibranch topology and the plain architecture, resulting in good speed–precision tradeoff in image classification. Zhang et al. [28] further extended RepVGG by combining multiple branches of different sizes and complexity to enrich feature spaces, including convolution sequences, multiscale convolution, and mean pools. Benefiting from the advantages of reparameterization, some works have successfully introduced it into SISR tasks. ECBSR [12] proposed an edge-oriented convolution block (ECB) that comprises four types of carefully designed operators to extract edge and texture details more effectively. SESR [13] designed a collapsible linear block (CLB), which consists of a series of linear convolutions that can be jumbled and merged in the inference phase. SESR achieved good balance between reconstruction image quality and computational complexity. However, as the depth of the network based on reparameterization has increased, problems such as slow training and overfitting have arisen. Therefore, we add residual connection and normalization to the multibranch structure to solve these problems.

### 2.3. Model Optimization

In computer vision tasks, a loss function is used to calculate and describe the gap between the prediction result and the ground truth, and this gap is quantified by the loss function to judge the degree of prediction error. Therefore, choosing an appropriate loss helps to obtain better results. Previous works on SISR tasks have tended to optimize network parameters through L1 and L2 losses [7]. However, some researchers found that using these losses alone may result in fuzzy and oversmoothed reconstructed images [14]. Therefore, a variety of special losses are proposed for SISR tasks. Feature reconstruction loss [29] was proposed to encourage the network to generate reconstructed images that are more similar to the ground truth in perception. The Laplacian pyramid network (LapSRN) [14] is applied the Charbonnier loss to improve the robustness of the deep SR network, which can better handle outliers. SRGAN [15] employed the perceptual loss, including content loss and adversarial loss, to make results more photorealistic. The u-shaped residual network (URNet) [30] uses a high-frequency loss design to alleviate the problem of oversmoothed SR images. In this work, we propose an efficient visual perceptual enhancement loss that is effective in improving the structural similarity of SR images.

## 3. Method

In this section, we first describe the overall structure of our proposed network. Then, we describe the structure of the reparameterizable multibranch bottleneck module and the process of reparameterization, respectively. Finally, we introduce the proposed PSE loss in detail, including the composition and the computation.

### 3.1. Network Structure

**Figure 1:**
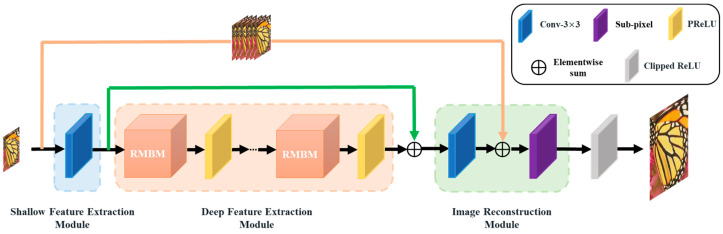
The training structure of the reparameterizable multibranch bottleneck network (t-RMBN).

**Figure 2:**
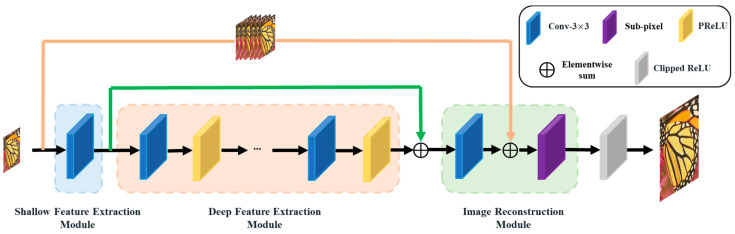
The deployment structure of the reparameterizable multibranch bottleneck network (d-RMBN).

As shown in Figure 1, the proposed reparameterizable multibranch bottleneck network (RMBN) consists of three parts: the shallow feature extraction module (SFEM), the deep feature extraction module (DFEM), and the image reconstruction module (IRM). SFEM is used to extract the shallow feature information of the input LR image. To further extract the rich high-frequency information, the shallow feature information is further parsed by a cascaded reparameterizable multibranch bottleneck module (RMBM) in DFEM. IRM processes the deep feature information to obtain the reconstructed SR image. The deployment network (d-RMBN), as illustrated in Figure 2, reduces the number of parameters and computation by replacing the RMBM with a simple 3 × 3 convolutional layer. It can be efficiently deployed for edge computing devices.

We use a 3 × 3 convolutional layer to extract shallow features. SFEM takes advantage of the fact that convolutional layers are good at extracting features to transform the LR image into high-dimensional shallow feature maps and filter out some of the low-frequency information. The process is expressed as follows:

(1) $$M_{0}=H_{SFEM}\left(I_{LR}\right),$$

where $I_{\mathrm{LR}}$ denotes the low-resolution (LR) image, and $H_{\mathrm{SFEM}}\;(\cdot )$ denotes the shallow feature extraction module. $M_{0}$ denotes the shallow feature maps.

The shallow feature maps are fed into the DFEM to extract deeper and more abstract high-level features in order to obtain high-frequency information. The process is expressed as follows:

(2) $$M_{DF}=H_{DFEM}\left(M_{0}\right),$$

where $H_{\mathrm{DFEM}}\;(\cdot )\;$denotes the deep feature extraction module, and $M_{\mathrm{DF}}\;$denotes the deep feature maps. DFEM consists of multiple cascaded reparameterizable multibranch bottleneck modules (RMBMs) and PReLU activation [31], which can be equivalently converted to cascaded 3 × 3 convolutional layers and PReLU activation in the deployment phase. The PReLU activation introduces an implicit nonlinearity to the RMBM module, allowing the optimization of the model to better achieve local minima and improve the stability and convergence of the training process. The RMBM can effectively extract detailed information for the SISR tasks and enhance the cross-channel learning capability of the network.

After the deep feature extraction module, we use IRM to fuse the shallow feature maps, deep feature maps, and image dimensional feature maps composed of LR images. We also perform upsampling operations by rearranging channel features into spatial dimensions using a subpixel convolution layer, which is expressed as follows:

(3) $$H_{IRM}=f_{up}\left(f_{3\times 3}\left(M_{DF}+M_{0}\right)+{\dot{I}}_{LR}\right),$$

(4) $$I_{SR}=H_{IRM}\left(M_{DF}+M_{0}+{\dot{I}}_{LR}\right),$$

where $H_{\mathrm{IRM}}\;(\cdot )\;$denotes the image reconstruction module, and $f_{\mathrm{up}}\;(\cdot )\;$denotes the sub-pixel convolutional layer function; $f_{3\times 3}\;(\cdot )$ denotes the 3 × 3 convolutional layer function, and ${\dot{I}}_{\mathrm{LR}}\;$denotes the image dimensional feature maps. $I_{\mathrm{SR}}\;$represents the output images.

The shallow feature maps mainly contain the low-frequency information represented by the background, while the deep feature maps include the high-frequency details, such as edges and contours required for the SR. The network can transfer low-frequency information directly to the IRM via residual learning of feature dimension and image dimension. This helps the DFEM to focus on recovering high-frequency information and to reduce the difficulty of network training.

SISR networks generally employ linear output of floating-point data with no data range constraints. These networks are prone to lose some important information when quantizing uint8, resulting in dull colors and severe degradation in the SR images. In this work, we add the constrained activation function (Clipped ReLU) to the output of the model and reduce performance degradation by restricting the output pixel values to the range 0–255. The process is expressed as follows:

(5) ClippedReLU(x)=max(0,min(x,255)),

where max (·) denotes the maximum value, and min (·) denotes the minimum value.

### 3.2. Reparameterizable Multibranch Bottleneck Module

**Figure 3:**
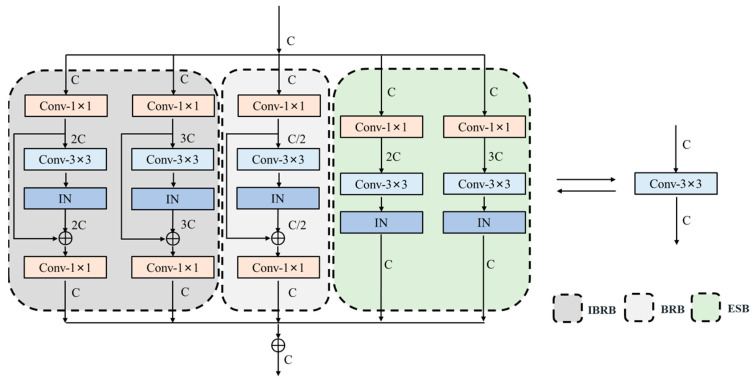
The structure of the reparameterizable multibranch bottleneck module (RMBM).

ECB [12] contains a single 3 × 3 convolutional layer branch and three edge detection operator branches. The edge detection operator branches are trained by predefined templates and given learnable scaling weights, which are equivalent to depthwise convolution and prolongs part of the training time. The learning ability of a single learnable scaling weight is insufficient and the extracted edge information is limited for complex scenes. The simple structure of a single 3 × 3 convolutional layer branch does not fully exploit the advantages of reparameterization. Inspired by ECB, we remove the these branches and design a new reparameterizable multibranch bottleneck module (RMBM) based on the reparameterization. Different from RepVGG [26] and the diverse branch block (DBB) [28], we introduce the bottleneck structure and the inverted bottleneck structure to the RMBM during the training phase, which allows the model to extract multiscale features by scaling the channel dimensions. Moreover, we replace the normalization in the structure, making the module more beneficial for SR tasks. As shown in Figure 3, RMBM is primarily composed of bottleneck residual blocks (BRB), inverted bottleneck residual blocks (IBRB), and expand–squeeze convolution blocks (ESB). It can extract edge and high-frequency texture details for SISR tasks more effectively, improve the feature representation capability, and shorten the training time for the network. Each component and its function are described below:

The BRB consists of two 1 × 1 convolutional layers, a 3 × 3 convolutional layer, and a residual connection. First, the 1 × 1 convolutional layer is employed to reduce the feature maps channel by half in order to achieve cross-channel interaction and information fusion. The 3 × 3 convolutional layer, which has a larger perceptual field than the 1 × 1 convolutional layer, is then utilized to extract the low-dimensional deep features. Finally, we apply the 1 × 1 convolutional layer to increase the feature maps. The BRB makes good use of the small number of parameters and low computational complexity of the 1 × 1 convolutional layer, and it improves the computational efficiency of the 3 × 3 convolutional layer while using the internal residual connection to effectively avoid gradient disappearance and explosion. The BRB can be expressed as follows:

(6) $$F_{BRB}=K_{S1}*\left[IN\left(K_{3}*\left(K_{F1}*X+B_{F1}\right)+\left(K_{F1}*X+B_{F1}\right)\right)\right]+B_{S1},$$

where ∗ denotes the convolution operation, and $K_{F1}{,\;B}_{F1\;}$, respectively, represent the weight and bias of the first 1 × 1 convolution layer. $K_{S1}{,\;B}_{S1}$, respectively, denote the weight and bias of the second 1 × 1 convolution layer, and $K_{3}\;$denotes the weight of the 3 × 3 convolution layer. IN (·) denotes the instance normalization, and $F_{\mathrm{BRB}}$ denotes the output of the BRB.

A wider range of features can significantly improve model representation and contribute to better performance on SISR tasks [32]. The reduced dimensionality of BRB may not be sufficient to retain sufficient high-frequency information. We design IBRB to expand the channel of the feature maps twice and three times using the first 1 × 1 convolutional layer, which enables the network to learn deeper features. IBRB improves the utilization of features and the representation capability of the network, and helps the information flow and gradient back-propagation of the network. The simple composition of IBRB and BRB reduces memory requirements and is suitable for most deep learning training frameworks, as well as being easily applied to edge computing devices. The process of IBRB is consistent with Equation (6).

Firstly, the feature maps channel is expanded to double and triple by a 1 × 1 convolution layer, which is compressed to the original by a 3 × 3 convolution layer to better learn the interrelationship between features. ESB further enhances the capability of feature extraction for RMBM. It can be expressed as follows:

(7) $$F_{ESB}=IN\left(K_{3}*\left(K_{1}*X+B_{1}\right)\right),$$

where $K_{1}{,\;B}_{1}$, respectively, denote the weight and bias of 1 × 1 convolutional layers. $K_{3}$ denotes the weight of 3 × 3 convolutional layers, and $F_{\mathrm{ESB}}$ denotes the output of the ESB.

After multibranch fusion, the final RMBM output can be expressed as follows:

(8) $$F=F_{BRB}+F_{IBRB}+F_{ESB},$$

As the network deepens, some models use batch normalization (BN) [33] to mitigate the covariance drift within the model. Owing to the variability in different image patches within each batch and the different configurations of training and testing, BN is not common in low-level vision tasks, especially for SISR tasks. It tends to produce block artifacts in SR results. Inspired by HINet [34], we add instance normalization (IN) [35] after the 3 × 3 convolutional layers in the above module to solve the overfitting problem caused by the overdepth network. IN also speeds the training and convergence of the network and prevents gradient explosion and disappearance. IN is a nonlinear operator in the training phase, which normalizes the feature mapping and contains learnable parameters to participate in the back-propagation computation. In the inference phase, IN becomes a linear operator that uses the parameters obtained during training to merge the 3 × 3 convolutional layers into a single 3 × 3 convolutional layer to reduce the number of parameters and the computational effort of the network.

### 3.3. Reparameterization

After the network training is completed, the RMBM can be reparameterized to equivalently transform the training model into a single-branch deployment model. The following describes the reparameterization method.

For ESB, the 1 × 1 expanded convolutional layer, 3 × 3 squeezed convolutional layer, and IN can be equivalently transformed into a single 3 × 3 convolutional layer by the following equations:

(9) $${\bar{K}}_{3}=\frac{\gamma }{\sqrt{\sigma^{2}+\epsilon }}\cdot K_{3},$$

(10) $${\bar{B}}_{3}=-\frac{\gamma \cdot \mu }{\sqrt{\sigma^{2}+\epsilon }}+\beta ,$$

(11) $$K_{n3}={\bar{K}}_{3}*perm\left(K_{1}\right),$$

(12) $$B_{n3}={\bar{K}}_{3}*rep\left(B_{1}\right)+{\bar{B}}_{3},$$

where μ, σ, γ, β denote the mean, variance, scale factor, and offset factor of IN, respectively; ϵ denotes the constant $10^{-5}$, and ${\bar{K}}_{3},\;{\bar{B}}_{3}$, respectively, denote the weight and bias of 3 × 3 convolutional layers after merging IN with 3 × 3 squeezed convolutional layers; perm (·) denotes the first and second dimensions of the exchange tensor $K_{1}$, and rep (·) denotes the broadcast operation; $K_{n3}{,\;B}_{n3}\;$denote the weight and bias of the 3 × 3 convolutional layers after merging the ESB.

**Figure 4:**
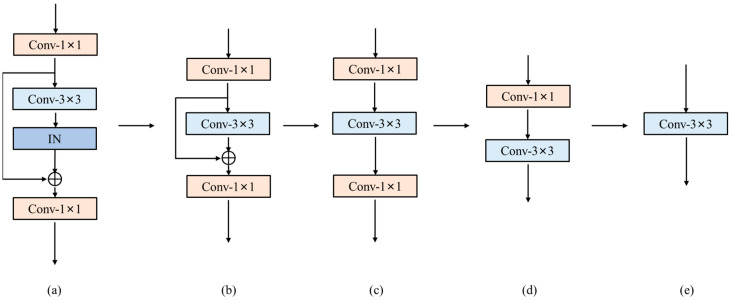
The process of reparameterization. The subfigures (**a**–**e**) describe how to reparameterize the BRB into a single 3 × 3 convolution.

For BRB and IBRB, the reparameterization process is shown in Figure 4. First, the transformation from (a) to (b) is achieved by repeating Equations (9)–(12) and combining the IN and the 3 × 3 convolutional layer into a single 3 × 3 convolutional layer. Then, the internal residual connection is replaced by a 3 × 3 convolutional layer with the center weight of the *i*-th channel of the *i*-th convolutional kernel being one and the rest being zero. Finally, the transformation from (b) to (c) is achieved by adding the weight and bias of the replaced 3 × 3 convolutional layer and the single 3 × 3 convolutional layer, respectively, which is calculated as follows:

(13) $$K_{3}={\bar{K}}_{3}+K_{r3},$$

(14) $$B_{3}={\bar{B}}_{3}+B_{r3},$$

where $K_{r3}{,\;B}_{r3}$ denote the weight and bias of the 3 × 3 convolutional layers replacing the residual connection; $\dot{K_{3}},\;\dot{B_{3}}$ denote the weight and bias of the 3 × 3 convolutional layers after merging the residual connection and the 3 × 3 convolutional layers, respectively. The transformation from (c) to (e) can be achieved by repeating the ESB equivalent transformation process twice.

After the above equivalent transformation, multibranches containing only a single 3 × 3 convolutional layer can be obtained. Since the multibranch module has only convolution before going through the PReLU activation [31], the five branches of convolutional layers can be combined into a single 3 × 3 convolutional layer by exploiting the additivity of convolution, which is computed as follows:

(15) $$K_{RMBM}=K_{1}+\ldots +K_{i}\;(i=1,2,\ldots ,5),$$

(16) $$B_{RMBM}=B_{1}+\ldots +B_{i}\;(i=1,2,\ldots ,5),$$

where $K_{i}{,\;B}_{i}$ denote the weight and bias of the *i*-th branch 3 × 3 convolutional layer, and $K_{\mathrm{RMBM}}{,\;B}_{\mathrm{RMBM}}$ denote the weight and bias of the 3 × 3 convolutional layer obtained from RMBM equivalent transformation.

### 3.4. Loss Function

Using only L1 or L2 loss leads to SR images that lack high-frequency detail and present unsatisfactory results with oversmoothed texture. Therefore, we propose a novel PSE loss, which consists of common objective evaluation metrics for SISR tasks (peak signal-to-noise ratio [36], structural similarity [37] and edge loss [38]). We design the loss function from the perspective of improving the evaluation metrics of the reconstructed images. Considering that the loss function always tends to be decreasing, we utilize the calculation of (1-SSIM) in the numerator and PSNR in the denominator to satisfy the requirement. During the training process, we found that there existed a very small value of PSNR, which made the calculation of (1-SSIM)/PSNR not appear as a number (NaN). Therefore, a value was needed to be added to the denominator to stabilize the loss calculation. Further, we found that adding a SSIM calculation can improve the metrics better than adding a tiny fixed value, especially for the SSIM metric. Then, inspired by [38], we also added edge loss to the final loss function to further enhance the model’s ability to extract edge features. It is simple to compute without additional learnable parameters, which are calculated as follows:

(17) $$L_{PSC}=\frac{1-SSIM(X,Y)}{PSNR(X,Y)+SSIM(X,Y)}+\alpha L_{Edge\;},$$

where X, Y denote the SR image and the ground truth, respectively; SSIM (·) denotes the calculated structural similarity, and PSNR (·) denotes the calculated peak signal-to-noise ratio; α denotes the weight parameter, which is empirically set to 0.05 to balance the loss term; $L_{\mathrm{Edge}}$ denotes the variant Charbonnier loss [14], which is calculated as follows:

(18) $$L_{Edge\;}=\sqrt{∥\Delta (X)-\Delta (Y){∥}^{2}+\varepsilon^{2}},$$

where Δ (·) is the Laplace operator, and ε denotes the constant $10^{-3}$.

## 4. Experiments

### 4.1. Datasets and Metrics

In this work, 3450 images from the DIV2K dataset [39] and Flicker2K [40] are used to train the network, and five standard benchmark datasets, including Set5 [41], Set14 [42], BSD100 [43], Urban100 [44], and DIV2K [39], are used to test the performance of the model. Various data augmentation methods are used to increase the size of the dataset during training, including random horizontal flip, random vertical flip, and random 90° rotation. In line with previous SISR algorithms, peak signal-to-noise ratio (PSNR) and structural similarity (SSIM) are employed as metrics to assess network performance. SR images are first converted from RGB to YCbCr color space, and the luminance component (Y) is taken to calculate the evaluation metric uniformly.

### 4.2. Implementation Details

We use the Adam [45] optimizer for training, where the optimizer parameters are $\beta_{1}=0.99{,\;\beta }_{2}=0.999,\;$and ${\varepsilon =10}^{-8}$. The training process is divided into two stages and the optimizer parameters are kept consistent. The first stage lasts 800 epochs, the learning rate is initialized to ${5\times 10}^{-4}$, and shrinks by half after every 200 epochs. In the first stage, 64 randomly cropped 64 × 64 patches from the LR images are used as the batch size for training. The L1 loss is used for the first stage of training. The second stage lasts for 200 epochs, and the network is trained using the proposed PSE loss based on the first stage pretraining model with a batch size of 16 image patches of 128 × 128. The learning rate is initialized to ${5\times 10}^{-4}$ and remains constant in the second stage. The networks are trained and tested on four NVIDIA GTX 3090 GPUs using the Pytorch 1.7.0 framework.

Similar to ECBSR [12], we set up RMBN with several groups of different sizes, including RMBN-M4C8, RMBN-M4C16, RMBN-M10C16, RMBN-M10C32, and RMBN-M16C64, where M denotes the number of RMBMs and C denotes the number of intermediate feature map channels.

### 4.3. Ablation Studies

To analyze the impact of the three blocks in RMBM, we conduct experiments on five standard benchmark datasets. The baseline network uses the modules containing five 3 × 3 convolutions without channel scaling operation. All models are trained from scratch using the same setting. As shown in Table 1, the performance of the base network can be improved by using any of the three components, and is further improved when the different components are stacked. When all three components are used simultaneously (0.32 dB improvement in PSNR value on the Set5 dataset), the performance reaches the highest. These results show that RMBM can make full use of the correlation between feature maps to facilitate the flow of information and make the network give more attention to the high-frequency texture details.

To verify the effectiveness of the constrained activation, two sets of comparison experiments are conducted to analyze the performance of the networks before and after quantization. We test the running time when the LR images are scaled to 1280 × 720 pixels on the edge computing device. As shown in Table 2, the PSNR value of the floating-point model decreases severely after uint8 quantization without using the constraint activation, especially on the Set14 dataset, which reaches 1.59 dB. However, the performance decreases by only 0.27 dB after using the constraint activation and increases only a few NPU running times (0.002 s). The experimental results show that constraint activation reduces the performance degradation caused by model quantization, making the SR results more suitable for network deployment.

As shown in Table 3, we design two sets of comparison experiments to prove the effectiveness of the feature dimension residual learning (FDRL). In addition, the performance of the prequantization and postquantization networks is also compared. We can see that the floating-point model with FDRL outperforms the one without it, and the NPU running time increases by only 0.001 s. The floating-point model without FDRL has significant performance degradation when quantizing uint8, which reaches 0.86 dB on the Set5 dataset. The experimental results show that FDRL can deliver more effective information deeper into the network and increase the usage of shallow features. FDRL can also reduce the loss of low-frequency information, and improve the model performance while reducing the degradation of performance during model quantization.

In order to verify the effectiveness of the PSE loss, we run comparison experiments between training all epochs with L1 or L2 loss only and training all epochs with combined L1 and PSE losses. As shown in Table 4, without any additional parameters, the PSE loss can improve the performance of the network. In particular, the SSIM metric increases the most, which is sensitive to human eyes, reaching 0.0038 on the Urban100 dataset. The results show that the proposed PSE loss takes the human visual system into account and significantly increases expression ability and network performance.

### 4.4. Comparison with State-of-the-Art Methods

In this work, we validate the proposed network SR performance on five standard benchmark datasets with upsampling scales of ×2 and ×4. The objective evaluation metrics and subjective visual effects of RMBN and some representative lightweight SISR models are compared, including bicubic; SRCNN [17]; fast super-resolution CNN (FSRCNN) [46]; ESPCN [19]; VDSR [18]; LapSRN [28]; fast, accurate, and lightweight super-resolution (FLASR) [47]; CARN-M [5]; IMDN [7]; ECBSR [12]; RFDN [8]; LatticeNet [25]; etc.

#### 4.4.1. Quantitative Results

Table 5 and Table 6 summarize the performance comparisons of different SISR networks on the five benchmark datasets. The implementation of the comparison algorithms is taken from the authors’ publicly available source code, and the comparison data used the results of other networks from published papers [16]. In addition to the PSNR/SSIM metrics, these tables also show the number of parameters and the computation for a more comprehensive comparison. The FLOPs are calculated when the images are upsampled to 1280 × 720 pixels.

**Figure 5:**
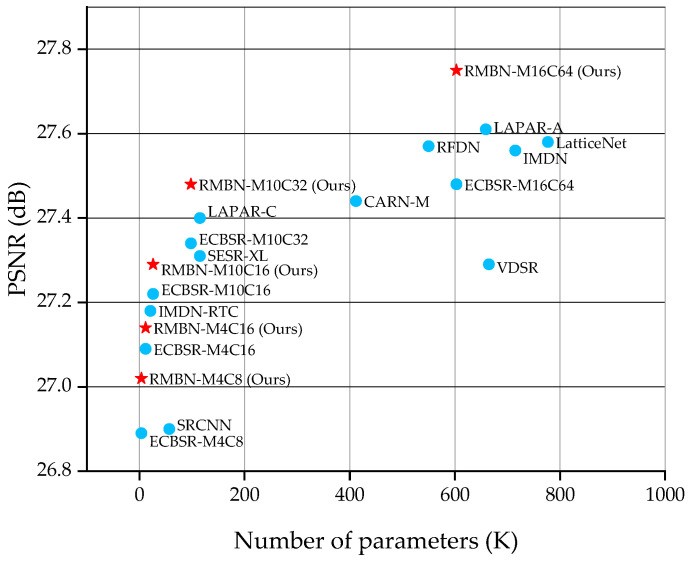
PSNR vs. the number of parameters. The comparison is conducted on the BSD100 dataset with the ×4 scale factor.

As shown in Table 5 and Table 6, our networks achieve the best objective evaluation metrics at all scales. RMBN-M4C8 is the smallest network in this work, which achieves better performance than SRCNN [17] and ESPCN [19], while reducing the number of parameters by a factor of 12 and 10, and the FLOPs by a factor of 92 and 9, respectively. RMBN-M4C8 and ECBSR-M4C8 [12] have the same number of parameters and computational effort, but we obtain higher objective evaluation metrics. Similarly, RMBN-M4C16, RMBN-M10C16, and RMBN-M10C32 significantly outperform other comparative networks, and the balance between the number of model parameters and FLOPs. In this work, we also compare RMBN extended to M16C64 with some more complex SISR networks, such as IMDN [7], LAPAR-A [24], and LatticeNet [25]. RMBN-M16C64 significantly reduces the computational complexity and has better performance. Compared with RFDN [8], RMBN-M16C64 achieves better performance with an average improvement of 0.1 dB in PSNR over the five benchmark datasets, which fully exploits the advantages of the reparameterization. In particular, the significant improvement on the Urban100 dataset, which contains richer structural texture information, demonstrates that the proposed network is able to reconstruct more texture details than other comparison networks. As shown in Figure 5, the proposed RMBN achieves better tradeoff between the performance of image SR and model complexity than other advanced lightweight models on the BSD100 dataset.

#### 4.4.2. Qualitative Results

**Figure 6:**
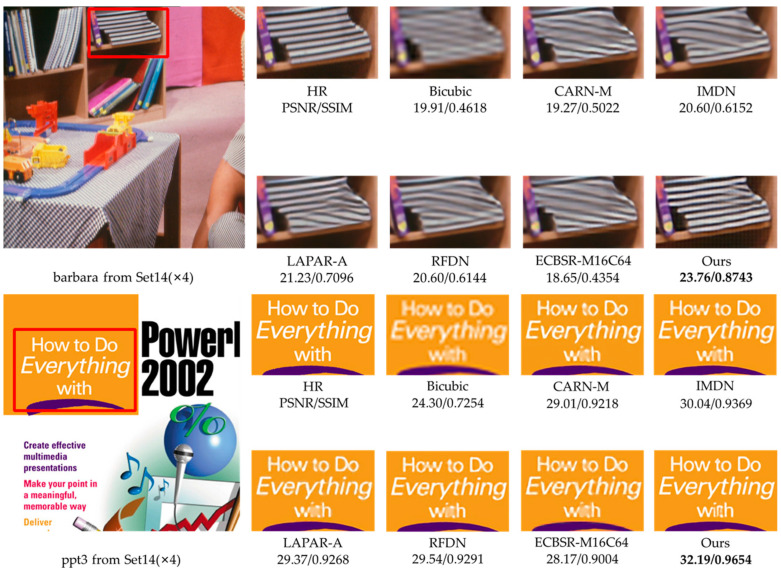
Visual qualitative comparisons of the lightweight methods on the Set14 dataset (×4 SR).

**Figure 7:**
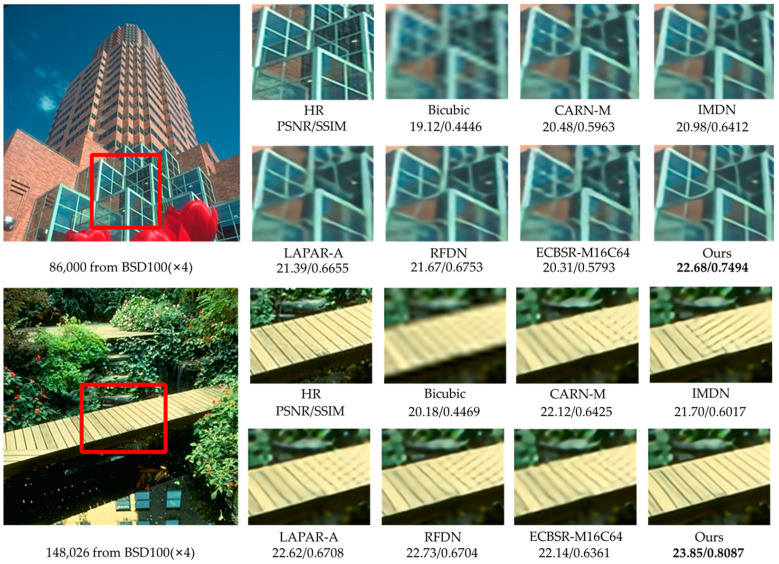
Visual qualitative comparisons of the lightweight methods on the BSD100 dataset (×4 SR).

**Figure 8:**
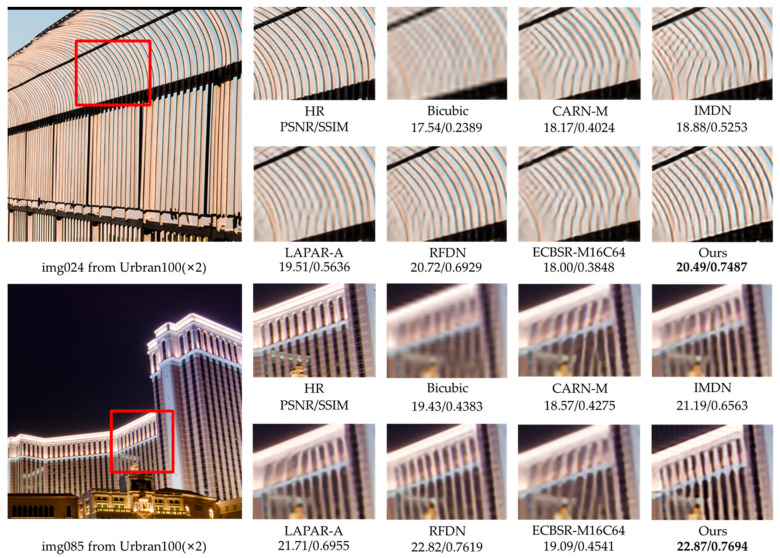
Visual qualitative comparisons of the lightweight methods on the Urban100 dataset (×2 SR).

**Figure 9:**
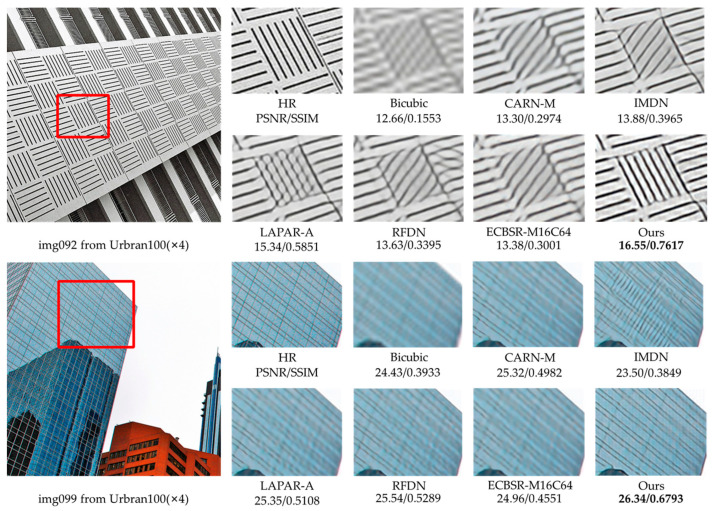
Visual qualitative comparisons of the lightweight methods on the Urban100 dataset (×4 SR).

Considering the effect of the parameter number on performance, we select RMBN-M16C64 to compare the ×2 and ×4 SR subjective visual effects with other lightweight SISR networks on the Set14, BSD100, and Urban100 datasets (see Figure 6, Figure 7, Figure 8 and Figure 9). The compared SR images are locally cropped and enlarged for observation.

It can be observed that most contrast networks produce blurred and inaccurate edge and texture details (see board stripes in Figure 7 and railing stripes in Figure 8), while RMBN can mitigate the ringing phenomenon and recover more accurate and sharper edge details. Some comparison networks (e.g., IMDN [7], ECBSR-M16C64 [12]) reconstruct images with the opposite texture orientation of the building as the ground truth, and even produce severe artifacts (see the book edges in Figure 6 and the target board stripes in Figure 9). While the RMBN-M16C64 correctly recovers the main structures, especially for regular structural patterns and text information, more high-frequency texture details are reconstructed, making the edges and contour features more visible (see the letters in Figure 6 and the glass window in Figure 9). These observations show that the proposed network is capable of recovering the edge information. The proposed PSE loss is used to improve the SR performance by fully considering the high-frequency texture information, solving the problem of oversmoothed SR images and enhancing the realism of results.

### 4.5. Edge Device Performance

We further test the running time of the network on edge devices since the number of parameters and FLOPs are unable to reflect the model’s inference speed. We also compare the performance of several representative lightweight SISR networks after uint8 quantization on the same device. As can be seen from Table 7 for scaling the image ×4 to 1280 × 720 pixels, common lightweight networks such as FSRCNN [46] and IMDN-RTC [7] fail to realize real-time inference on the edge devices. Furthermore, RMBN-M10C32 achieves real-time image SR with the lowest guaranteed accuracy loss and the highest evaluation metrics (running time of 0.032 s) after model quantization. Compared to RFDN [8], which utilizes an attention mechanism, the proposed network achieves better performance while reducing the inference time by a factor of five. It demonstrates that a network containing only 3 × 3 convolutional layers and activation is more suitable for deployment in edge computing devices. Compared to ECBSR-M10C32 [12] with the same model size, RMBN-M10C32 only increases the running time slightly, but obtains a 0.97 dB improvement in PSNR value on the Set14 dataset. These results show that RMBN achieves good balance between performance, parameters, and computational complexity, which is more favorable for network deployment.

### 4.6. Remote Sensing Image Super-Resolution

Since the remote sensing images have complex scenes and massive background information, more attention to useful information is needed in SR reconstruction [48]. Moreover, remote sensing images are generally high resolution, making SR reconstruction more difficult.

To demonstrate the effectiveness of the proposed network, we train and test it on public remote sensing datasets. UC-Merced [49] is a remote sensing image dataset used for land-use research, with 21 categories, each with 1000 images of 256 × 256 pixels. We randomly select 40 images from each category, obtain LR images by bicubic downsampling, and use these 840 pairs of images as the training dataset. The NWPU45 [50] dataset is a large-scale public dataset for remote sensing image scene classification, containing 45 categories of scenes, and the sample of each category contains 700 images with 256 × 256 pixels. AID [51] is an aerial image dataset, which consists of 30 types of aerial scenes, with 10,000 images in each scene. The above datasets have the characteristics of large scale and rich information. We randomly select 100 remote sensing images from the NWPU45 and AID datasets, respectively, and also use bicubic downsampling to obtain LR images for testing the network. We fine-tune our model with the proposed PSE loss based on the natural image training, which lasts 200 epochs with a learning rate of ${5\times 10}^{-4}$ and a batch size of 64. To ensure fairness, we use the same training strategy to fine-tune the comparison algorithms for training remote sensing images as their papers.

Table 8 shows the quantitative results of the representative SR methods on remote sensing datasets. We can notice that the proposed RMBN-M16C64 has the highest PSNR and SSIM on these two datasets, with an average PSNR improvement of 0.15 dB over RFDN [8]. The low-level feature information from the natural image dataset is allowed to be shared with the remote sensing datasets by employing the pretraining strategy, resulting in better performance. The results of the experiments reveal that our network is more general and capable of capturing useful information in complex backgrounds. Using the PSE loss, the network can effectively extract texture details from remote sensing images and obtain SR images with higher quantitative metrics.

**Figure 10:**
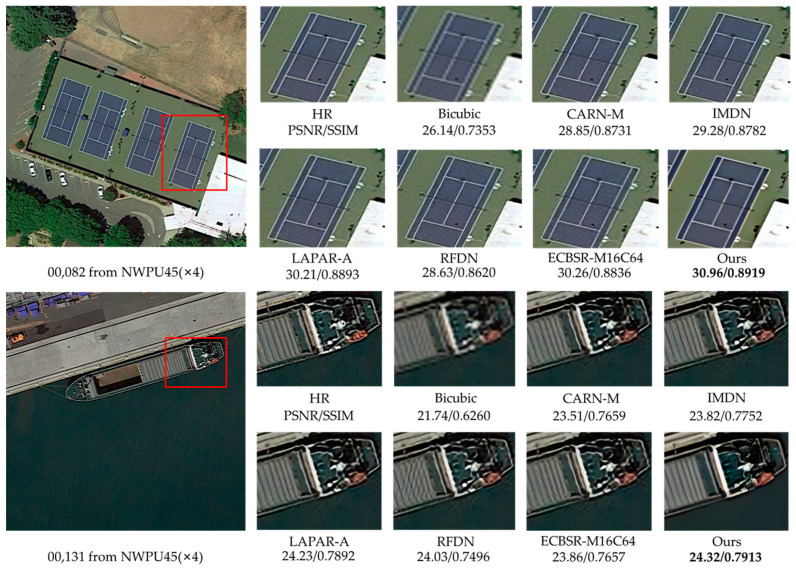
Visual qualitative comparisons of the lightweight methods on the NWPU45 dataset (×4 SR).

**Figure 11:**
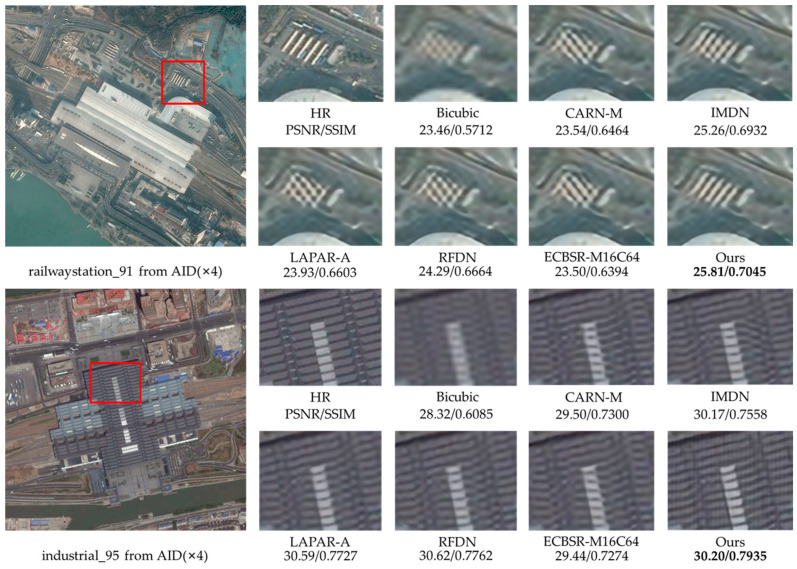
Visual qualitative comparisons of the lightweight methods on the AID dataset (×4 SR).

To fully demonstrate the effectiveness of our network, we also show the ×4 SR visual results for the NWPU45 and AID datasets in Figure 10 and Figure 11. It can be observed that our network is more advantageous in recovering remote sensing images with more texture details, especially for lines and repetitive structures (see the court in Figure 10 and the top of the building in Figure 11). Other contrast algorithms are prone to produce artifacts and blending when recovering remote sensing images with complex backgrounds, while our network can effectively reduce the blur (see the boat in Figure 10 and the house in Figure 11) and reconstruct more edge details.

## 5. Conclusions

In this work, we propose a new lightweight SISR network named RMBN to solve the problems of high computational complexity and large model size of existing CNN-based SISR algorithms for edge computing devices. Specifically, we design a reparameterizable multibranch bottleneck module (RMBM) to separate the training phase from the deployment phase by using the reparameterization. In the training phase, RMBM can fully utilize and fuse features of different widths and depths based on the multibranch structure. In the inference phase, the RMBM is collapsed by reparameterization, which reduces the number of parameters while increasing the inference speed. In addition, we propose a novel PSE loss for SISR tasks, making the network focus on recovering high-frequency details while alleviating the problem of oversmoothed images. Numerous experimental results show that the proposed network can improve visual perception and enhance high-frequency information such as edges and textures. By using constrained activation, the network significantly reduces performance degradation when deployed to edge computing devices. In comparison to advanced algorithms, RMBN achieves a better balance of reconstruction performance, model complexity, and inference speed. In the future, we intend to further reduce the size and calculation complexity of the training model to reduce the training time of the network.

## Figures and Tables

**Table 1:** Ablation experiment results of different blocks based on RMBN-M4C16 (×4 SR). **Bold** indicates the best performance.

Module			Set5 PSNR/SSIM	Set14 PSNR/SSIM	BSD100 PSNR/SSIM	Urban100 PSNR/SSIM	DIV2K PSNR/SSIM
BRB	IBRB	ESB					
×	×	×	30.82/0.8769	27.58/0.7680	27.00/0.7272	24.42/0.7435	29.38/0.8188
√	×	×	30.89/0.8781	27.62/0.7683	27.04/0.7279	24.47/0.7440	29.41/0.8195
×	√	×	31.08/0.8795	27.71/0.7690	27.09/0.7286	24.62/0.7451	29.55/0.8212
×	×	√	31.01/0.8790	27.66/0.7687	27.08/0.7282	24.53/0.7443	29.48/0.8201
√	×	√	31.10/0.8799	27.74/0.7694	27.10/0.7289	24.66/0.7458	29.60/0.8214
√	√	×	31.12/0.8804	27.77/0.7708	27.11/0.7291	24.72/0.7462	29.63/0.8218
×	√	√	31.13/0.8806	27.80/0.7714	27.12/0.7292	24.77/0.7464	29.65/0.8220
√	√	√	**31.14/0.8810**	**27.82/0.7721**	**27.14/0.7295**	**24.83/0.7469**	**29.69/0.8222**

**Table 2:** Ablation experiment results of constraint activation based on RMBN-M4C16 (×4 SR).

Clipped ReLU	Model-Type	Set5 PSNR/SSIM	Set14 PSNR/SSIM	Running Time (s)
**×**	fp32	31.14/0.8810	27.82/0.7721	0.018
	uint8	29.96/0.8801	26.23/0.7710	
√	fp32	31.14/0.8810	27.82/0.7721	0.020
	uint8	30.87/0.8808	27.55/0.7717	

**Table 3:** Ablation experiment results of FDRL based on RMBN-M4C16 (×4 SR).

FDRL	Model-Type	Set5 PSNR/SSIM	Set14 PSNR/SSIM	Running Time (s)
×	fp32	31.06/0.8808	27.80/0.7716	0.019
	uint8	30.22/0.8802	26.98/0.7709	
√	fp32	31.14/0.8810	27.82/0.7721	0.020
	uint8	30.87/0.8808	27.55/0.7717	

**Table 4:** Ablation experiment results of loss based on RMBN-M4C16 (×4 SR). **Bold** indicates the best performance.

Loss	Set5 PSNR/SSIM	Set14 PSNR/SSIM	BSD100 PSNR/SSIM	Urban100 PSNR/SSIM	DIV2K PSNR/SSIM
L1	31.08/0.8807	27.80/0.7704	27.10/0.7286	24.82/0.7431	29.66/0.8200
L2	31.02/0.8801	27.74/0.7698	27.03/0.7277	24.71/0.7428	29.60/0.8196
L1 + L_PSE_	**31.14/0.8810**	**27.82/0.7721**	**27.14/0.7295**	**24.83/0.7469**	**29.69/0.8222**

**Table 5:** Average performance of the lightweight methods (×2 SR). Best and second-best results are **bolded** and underlined.

Method	Scale	Params (K)	FLOPs (G)	Set5 PSNR/SSIM	Set14 PSNR/SSIM	BSD100 PSNR/SSIM	Urban100 PSNR/SSIM	DIV2K PSNR/SSIM
bicubic	×2	-	-	33.68/0.9307	30.24/0.8693	29.56/0.8439	26.88/0.8408	32.45/0.9043
SRCNN [17]		24.00	52.70	36.66/0.9542	32.42/0.9063	31.36/0.8879	29.50/0.8946	34.61/0.9334
ESPCN [19]		21.18	4.55	36.83/0.9564	32.40/0.9096	31.29/0.8917	29.48/0.8975	34.63/0.9342
ECBSR-M4C8 [12]		2.80	0.64	36.93/0.9577	32.51/0.9107	31.44/0.8932	29.68/0.9014	34.80/0.9356
RMBN-M4C8 (Ours)		2.80	0.64	**37.01/0.9580**	**32.62/0.9114**	**31.48/0.8936**	**29.72/0.9022**	**34.88/0.9366**
FSRCNN [46]	×2	12.46	6.00	36.98/0.9556	32.62/0.9087	31.50/0.8904	29.85/0.9009	34.74/0.9340
SESR-M5 [13]		13.52	3.11	37.39/0.9585	32.84/0.9115	31.70/0.8938	30.33/0.9087	35.24/0.9389
ECBSR-M4C16 [12]		10.20	2.34	37.33/0.9593	32.81/0.9129	31.66/0.8961	30.31/0.9091	35.15/0.9382
RMBN-M4C16 (Ours)		10.20	2.34	**37.40/0.9588**	**32.88/0.9136**	**31.77/0.8969**	**30.44/0.9106**	**35.26/0.9391**
IMDN-RTC [7]	×2	19.70	4.57	37.51/0.9600	32.93/0.9144	31.79/0.8980	30.67/0.9140	35.34/0.9398
SESR-M11 [13]		27.34	6.30	37.58/0.9593	33.03/0.9128	31.85/0.8956	30.72/0.9136	35.45/0.9404
ECBSR-M10C16 [12]		24.30	5.60	37.55/0.9602	32.98/0.9144	31.85/0.8985	30.78/0.9149	35.38/0.9402
RMBN-M10C16 (Ours)		24.30	5.60	**37.61/0.9609**	**33.03/0.9146**	**31.88/0.8989**	**31.02/0.9156**	**35.51/0.9406**
LapSRN [14]	×2	813.00	29.90	37.52/0.9590	33.08/0.9130	31.80/0.8950	30.41/0.9100	35.31/0.9400
FLASR-C [47]		408.00	93.70	37.66/0.9586	33.26/0.9140	31.96/0.8965	31.24/0.9187	35.57/0.9407
SESR-XL [13]		105.37	24.27	37.77/0.9601	33.24/0.9145	31.99/0.8976	31.16/0.9184	35.67/0.9420
ECBSR-M10C32 [12]		94.70	21.81	37.76/0.9609	33.26/0.9146	32.04/0.8986	31.25/0.9190	35.68/0.9421
LAPAR-C [24]		87.00	35.00	37.65/0.9593	33.20/0.9141	31.95/0.8969	31.10/0.9178	35.54/0.9411
RMBN-M10C32 (Ours)		94.70	21.81	**37.80/0.9611**	**33.31/0.9152**	**32.10/0.9006**	**31.34/0.9201**	**35.73/0.9427**
VDSR [18]	×2	665.00	612.60	37.53/0.9587	33.05/0.9127	31.90/0.8960	30.77/0.9141	35.43/0.9410
CARN-M [5]		412.00	91.20	37.53/0.9583	33.26/0.9141	31.92/0.8960	31.23/0.9193	35.62/0.9420
ECBSR-M16C64 [12]		596.00	137.31	37.90/0.9615	33.34/0.9178	32.10/0.9018	31.71/0.9250	35.79/0.9430
IMDN [7]		694.00	158.80	38.00/0.9605	33.63/0.9177	32.19/0.8996	32.17/0.9283	35.87/0.9436
LAPAR-A [24]		548.00	171.00	38.01/0.9605	33.62/0.9183	32.19/0.8999	32.10/0.9283	35.89/0.9438
RFDN [8]		534.00	95.00	38.05/0.9606	33.68/0.9184	32.16/0.8994	32.12/0.9278	35.80/0.9433
LatticeNet [25]		756.00	169.50	38.06/0.9607	33.70/0.9187	32.20/0.8999	32.25/0.9288	35.88/0.9436
RMBN-M16C64 (Ours)		596.00	137.31	**38.16/0.9621**	**33.74/0.9192**	**32.27/0.9034**	**32.29/0.9296**	**35.94/0.9444**

**Table 6:** Average performance of the lightweight methods (×4 SR). Best and second-best results are **bolded** and underlined.

Method	Scale	Params (K)	FLOPs (G)	Set5 PSNR/SSIM	Set14 PSNR/SSIM	BSD100 PSNR/SSIM	Urban100 PSNR/SSIM	DIV2K PSNR/SSIM
bicubic	×4	-	-	28.43/0.8113	26.00/0.7025	25.96/0.6682	23.14/0.6577	28.10/0.7745
SRCNN [17]		57.00	52.70	30.48/0.8628	27.49/0.7503	26.90/0.7101	24.52/0.7221	29.25/0.8090
ESPCN [19]		24.90	1.44	30.52/0.8697	27.42/0.7606	26.87/0.7216	24.39/0.7241	29.32/0.8120
ECBSR-M4C8 [12]		3.70	0.21	30.52/0.8698	27.43/0.7608	26.89/0.7220	24.41/0.7263	29.35/0.8133
RMBN-M4C8 (Ours)		3.70	0.21	**30.61/0.8706**	**27.50/0.7614**	**27.02/0.7223**	**24.55/0.7289**	**29.40/0.8141**
FSRCNN [46]	×4	12.00	5.00	30.70/0.8657	27.59/0.7535	26.96/0.7128	24.60/0.7258	29.36/0.8110
SESR-M5 [13]		18.32	1.05	30.99/0.8764	27.81/0.7624	27.11/0.7199	24.80/0.7389	29.65/0.8189
ECBSR-M4C16 [12]		11.90	0.69	31.04/0.8805	27.78/0.7693	27.09/0.7283	24.79/0.7422	29.62/0.8197
RMBN-M4C16 (Ours)		11.90	0.69	**31.14/0.8810**	**27.82/0.7721**	**27.14/0.7295**	**24.83/0.7469**	**29.69/0.8222**
IMDN-RTC [7]	×4	21.00	1.22	31.22/0.8844	27.92/0.7730	27.18/0.7314	24.98/0.7504	29.76/0.8230
SESR-M11 [13]		32.14	1.85	31.27/0.8810	27.94/0.7660	27.20/0.7225	25.00/0.7466	29.81/0.8221
ECBSR-M10C16 [12]		26.00	1.50	31.37/0.8866	27.99/0.7740	27.22/0.7329	25.08/0.7540	29.80/0.8241
RMBN-M10C16 (Ours)		26.00	1.50	**31.41/0.8876**	**28.10/0.7753**	**27.29/0.7338**	**25.09/0.7549**	**29.92/0.8252**
LapSRN [14]	×4	813.00	149.40	31.54/0.8850	28.19/0.7720	27.32/0.7280	25.21/0.7560	29.88/0.8250
SESR-XL [13]		114.97	6.62	31.54/0.8866	28.12/0.7712	27.31/0.7277	25.31/0.7604	29.94/0.8266
ECBSR-M10C32 [12]		98.10	5.65	31.66/0.8911	28.15/0.7776	27.34/0.7363	25.41/0.7653	29.98/0.8281
LAPAR-C [24]		115.00	25.00	31.72/0.8884	28.31/0.7718	27.40/0.7292	25.49/0.7651	30.01/0.8284
RMBN-M10C32 (Ours)		98.10	5.65	**31.79/0.8912**	**28.37/0.7780**	**27.48/0.7372**	**25.54/0.7665**	**30.04/0.8289**
VDSR [18]	×4	665.00	612.60	31.35/0.8838	28.02/0.7678	27.29/0.7252	25.18/0.7525	29.82/0.8240
CARN-M [5]		412.00	46.10	31.92/0.8903	28.42/0.7762	27.44/0.7304	25.62/0.7694	30.10/0.8311
ECBSR-M16C64 [12]		602.90	34.73	31.92/0.8946	28.34/0.7817	27.48/0.7393	25.81/0.7773	30.15/0.8315
IMDN [7]		715.00	40.90	32.21/0.8948	28.58/0.7811	27.56/0.7353	26.04/0.7838	30.22/0.8336
LAPAR-A [24]		659.00	94.00	32.15/0.8944	28.61/0.7818	27.61/0.7366	26.14/0.7871	30.24/0.8346
RFDN [8]		550.00	23.90	32.24/0.8952	28.61/0.7819	27.57/0.7360	26.11/0.7858	30.26/0.8344
LatticeNet [25]		777.00	43.60	32.21/0.8943	28.61/0.7812	27.57/0.7355	26.14/0.7844	30.26/0.8348
RMBN-M16C64 (Ours)		602.90	34.73	**32.28/0.8957**	**28.66/0.7829**	**27.75/0.7408**	**26.24/0.7886**	**30.28/0.8350**

**Table 7:** Performance comparisons of the lightweight methods on RKNPU. **Bold** indicates the best performance.

Scale	Method	Set5 PSNR-uint8	Set14 PSNR-uint8	Running Time (s)
×2	bicubic	33.68	30.24	**0.025**
	FSRCNN [46]	34.66	30.17	0.074
	IMDN-RTC [7]	36.03	30.89	0.172
	SESR-XL [13]	36.14	31.45	0.057
	ECBSR-M10C32 [12]	36.22	31.91	0.045
	RFDN [8]	37.48	32.56	0.211
	RMBN-M10C32 (Ours)	**37.56**	**33.17**	0.049
×4	bicubic	28.43	26.00	**0.020**
	FSRCNN [46]	29.01	26.47	0.042
	IMDN-RTC [7]	30.04	26.88	0.120
	SESR-XL [13]	30.66	27.01	0.038
	ECBSR-M10C32 [12]	30.98	27.04	0.027
	RFDN [8]	31.12	27.49	0.178
	RMBN-M10C32 (Ours)	**31.42**	**28.01**	0.032

**Table 8:** Average performance of lightweight methods on the NWPU45 and AID datasets (×4 SR). Best and second-best results are **bolded** and underlined.

Scale	Method	NWPU45 PSNR/SSIM	AID PSNR/SSIM
×4	Bicubic	29.12/0.7494	26.09/0.7154
	CARN-M [5]	30.25/0.7845	27.32/0.7678
	ECBSR-M16C64 [12]	30.20/0.7828	27.31/0.7660
	IMDN [7]	30.37/0.7861	27.41/0.7704
	LAPAR-A [24]	30.42/0.7877	27.47/0.7726
	RFDN [8]	30.46/0.7889	27.51/0.7736
	RMBN-M16C64 (Ours)	**30.61/0.7900**	**27.67/0.7754**

## Data Availability

Publicly available datasets were analyzed in this study. Our training set DIV2K can be obtained from: https://data.vision.ee.ethz.ch/cvl/DIV2K/ (accessed on 18 October 2021). The URLs of test sets Set5, Set14, BSD100, and Urban 100 are available online at: https://sites.google.com/site/romanzeyde/research-interests (accessed on 18 October 2021); https://www.eecs.berkeley.edu/Research/Projects/CS/vision/bsds/ (accessed on 18 October 2021); https://sites.google.com/site/jbhuang0604/publications/struct_sr (accessed on 18 October 2021). The super-resolution algorithm codes are available online at: https://github.com/zwh001zwh/RMBN (accessed on 10 March 2023).
